# Crystal structure of γ-methyl l-glutamate *N*-carb­oxy anhydride

**DOI:** 10.1107/S2056989014026917

**Published:** 2015-01-01

**Authors:** Hitoshi Kanazawa, Aya Inada, Aya Sakon, Hidehiro Uekusa

**Affiliations:** a Faculty of Symbiotic Systems Science, Fukushima University, Kanayagawa-1, Fukushima 960-1296, Japan; b Chemistry and Materials Science, Tokyo Institute of Technology, Ookayama-2, Meguro-ku, Tokyo 152-8551, Japan

**Keywords:** crystal structure, polymerization, amino acid *N*-carb­oxy anhydrides, hydrogen bonding

## Abstract

In the title compound, C_7_H_9_NO_5_, alternative name *N*-carb­oxy-l-glutamic anhydride γ-methyl ester, the oxazolidine ring is essentially planar with a maximum deviation of 0.020 (3) Å. In the crystal, mol­ecules are linked by N—H⋯O hydrogen bonds between the imino group and the carbonyl O atom in the methyl ester group, forming a tape structure along the *a-*axis direction. The tapes are linked by C—H⋯O inter­actions into a sheet parallel to the *ac* plane. The tapes are also stacked along the *b* axis with short contacts between the oxazolidine rings [C⋯O contact distances = 2.808 (4)–3.060 (4) Å], so that the oxazolidine rings are arranged in a layer parallel to the *ab* plane. This arrangement of the oxazolidine rings is very preferable for the polymerization of the title compound in the solid state.

## Abstract 2

Solid-state polymerization behavior of amino acid *N*-carb­oxy anhydrides is explained by the very preferable mol­ecular arrangement for the reaction in the crystal structure.

## Chemical context   

*N*-Carb­oxy anhydrides (NCAs) of amino acids are crystalline compounds and are polymerized in solution to prepare poly(amino­acid)s (Kricheldorf, 2006 ▸). Although amino acid NCAs are easily soluble in usual polar organic solvents such as tetra­hydro­furan, ethyl­acetate and 1,4-dioxane, *etc*., usual poly(amino­acid)s such as poly(l-alanine) and poly(l-valine) are not soluble in them. Thus, the solution polymerization of amino acid NCAs does not proceed in a real solution state but in a heterogeneous state. When amino acid NCA crystals are dipped in hexane (an inactive solvent) and butyl­amine is added to the mixture, polymerization takes place in the solid state. We have studied this solid state polymerization and found that the polymerization is quite different in each amino acid NCA. In addition, we found the solid-state polymerization is available for any amino acid NCAs for which solution polymerization is impossible.
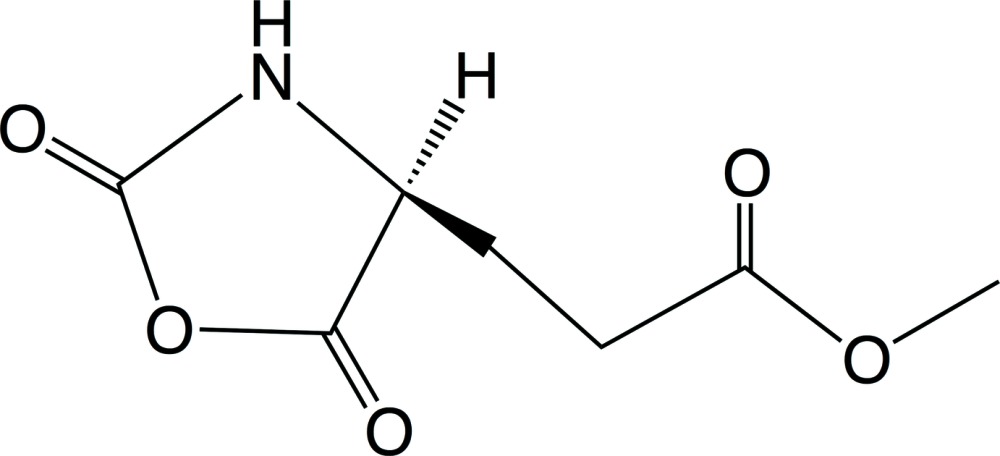


We have reported the crystal structures of glycine NCA (Kanazawa *et al.*, 1976*a* ▸) and l-alanine NCA (Kanazawa *et al.*, 1976*b* ▸), and found the polymerization rate depended on the crystal structure (Kanazawa & Kawai, 1980 ▸). We found that l-leucine NCA was the most reactive in the solid state polymerization among the examined amino acid NCAs, and the solution polymerization reactivity of l-alanine NCA in aceto­nitrile seemed to be more reactive than that in the solid state. However, when well-purified l-alanine NCA crystals were polymerized in aceto­nitrile solution or the solid state under strict moisture-free conditions, the reactivity in the solid state seemed similar to that in aceto­nitrile (Kanazawa *et al.*, 2006 ▸). The title compound (MLG NCA), (I), was very reactive in the solid state among the examined NCAs, and the conformation of the resulting poly(MLG) was mainly the β structure, while the poly(MLG) obtained in the solution reaction is the α helix. This high reactivity and the difference in the mol­ecular conformation of resulting polymer in the solid state are considered to be caused by the mol­ecular arrangement in the crystal of MLG NCA. Therefore, it is important to determine the crystal structure. Herein, we present the crystal and mol­ecular structure of (I).

## Structural commentary   

**Figure 1:**
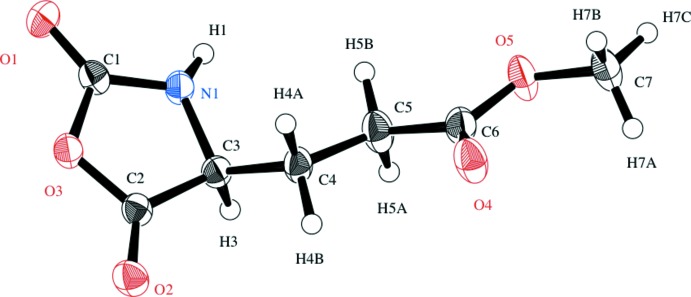
The mol­ecular structure of the title compound with the atom-numbering scheme. Displacement ellipsoids are drawn at the 50% probability level and H atoms are shown as spheres of arbitrary radii.

The atom-numbering scheme is shown in Fig. 1 ▸. The oxazolidine ring is essentially planar with a maximum deviation of 0.020 (3) Å

## Supra­molecular features   

**Figure 2:**
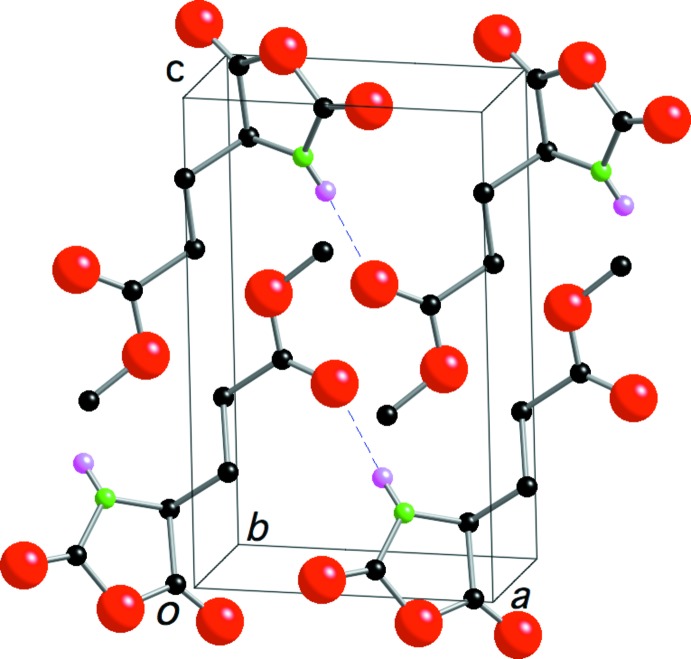
A packing diagram of the title compound viewed approximately along the *b* axis. N—H⋯O hydrogen bonds are shown as dashed lines. H atoms not involved in the hydrogen bonds have been omitted.

In the crystal structure (Fig. 2 ▸), MLG NCA mol­ecules are linked by N1—H1⋯O4^i^ hydrogen bonds (Table 1 ▸), forming a tape structure along the *a-*axis direction. The tapes are linked by C7—H7*A*⋯O2^ii^ inter­actions into a sheet parallel to the *ac* plane. The tapes are also stacked along the *b* axis with short contacts between the oxazolidine rings [C⋯O contact distances = 2.808 (4)–3.060 (4) Å], so that the oxazolidine rings are arranged in a layer parallel to the *ab* plane. As seen in Fig. 2 ▸, the five-membered rings in (I) are packed in one layer, and the –CH_2_CH_2_COOCH_3_ groups are packed in another layer, and the two layers are stacked alternately. This sandwich structure is one of the important requirements for high reactivity in the solid state, because the five-membered rings can react with each other within the layer. In the crystal, MLG NCA mol­ecules are considered to be polymerized and poly(MLG) with the β structure is formed.

## Synthesis and crystallization   

The synthesis of γ-methyl-l-glutamate (MLG) was carried out by the reaction of l-glutamic acid with methanol similarly to BLG. Compound (I) was obtained by the reaction of γ-methyl-l-glutamate with tri­chloro­methyl chloro­formate or triphosgene in tetra­hydro­furan, as reported previously for β-benzyl-l-aspartate NCA (Kanazawa & Magoshi, 2003 ▸). The reaction product was recrystallized in a mixture of ethyl­acetate and hexane (1:50 *v*/*v*), avoiding moisture contamination.

## Refinement details   

Crystal data, data collection and structure refinement details are summarized in Table 2 ▸. C-bound H atoms were included in calculated positions (C—H = 0.98–1.00 Å) and treated as riding, with *U*_iso_(H) = 1.2*U*_eq_(C) or 1.5*U*_eq_(C_meth­yl_). The H atom of the NH group was found in a difference Fourier map and was refined with *U*_iso_(H) = 1.2*U*_eq_(N).

## Supplementary Material

Crystal structure: contains datablock(s) global, I. DOI: 10.1107/S2056989014026917/is5376sup1.cif

Structure factors: contains datablock(s) I. DOI: 10.1107/S2056989014026917/is5376Isup2.hkl

Supporting information file. DOI: 10.1107/S2056989014026917/is5376Isup4.mol

Supporting information file. DOI: 10.1107/S2056989014026917/is5376Isup4.cml

CCDC reference: 1038016

Additional supporting information:  crystallographic information; 3D view; checkCIF report

## Figures and Tables

**Table 1:** Hydrogen-bond geometry (, )

*D*H*A*	*D*H	H*A*	*D* *A*	*D*H*A*
N1H1O4^i^	0.86(3)	2.07(3)	2.926(3)	176(3)
C7H7*A*O2^ii^	0.98	2.54	3.366(4)	142

Symmetry codes: (i) 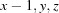; (ii) 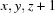.

**Table 2:** Experimental details

Crystal data	
Chemical formula	C_7_H_9_NO_5_
*M*_r_	187.15
Crystal system, space group	Monoclinic, *P*2_1_
Temperature (K)	123
*a*, *b*, *c* ()	6.0101(4), 7.1760(5), 9.8528(6)
()	93.190(4)
*V* (^3^)	424.28(5)
*Z*	2
Radiation type	Cu *K*
(mm^1^)	1.10
Crystal size (mm)	0.16 0.06 0.05

Data collection	
Diffractometer	Rigaku R-AXIS RAPID-II
Absorption correction	Multi-scan (*ABSCOR*; Higashi, 1995 ▸)
*T*_min_, *T*_max_	0.844, 0.947
No. of measured, independent and observed [*F* ^2^ > 2(*F* ^2^)] reflections	4941, 1533, 1249
*R*_int_	0.060
(sin /)_max_ (^1^)	0.602

Refinement	
*R*[*F* ^2^ > 2(*F* ^2^)], *wR*(*F* ^2^), *S*	0.042, 0.088, 1.04
No. of reflections	1533
No. of parameters	121
No. of restraints	1
H-atom treatment	H atoms treated by a mixture of independent and constrained refinement
_max_, _min_ (e ^3^)	0.19, 0.19
Absolute structure	Flack *x* determined using 421 quotients [(*I* ^+^)(*I* )]/[(*I* ^+^)+(*I* )] (Parsons *et al.*, 2013 ▸)
Absolute structure parameter	0.08(19)

Computer programs: *RAPID-AUTO* (Rigaku, 2004 ▸), *SHELXS97* and *SHELXL2014* (Sheldrick, 2008 ▸), CrystalMaker (*CrystalMaker*, 2013 ▸) and *CrystalStructure* (Rigaku, 2010 ▸).

## supplementary crystallographic information

### Crystal data

**Table:**

C_7_H_9_NO_5_	*F*(000) = 196
*M**_r_* = 187.15	*D*_x_ = 1.465 Mg m^−^^3^
Monoclinic, *P*2_1_	Cu *K*α radiation, λ = 1.54186 Å
*a* = 6.0101 (4) Å	Cell parameters from 4941 reflections
*b* = 7.1760 (5) Å	θ = 4.5–68.1°
*c* = 9.8528 (6) Å	µ = 1.10 mm^−^^1^
β = 93.190 (4)°	*T* = 123 K
*V* = 424.28 (5) Å^3^	Column, colorless
*Z* = 2	0.16 × 0.06 × 0.05 mm

### Data collection

**Table:**

Rigaku R-AXIS RAPID-II diffractometer	1533 independent reflections
Radiation source: fine-focus rotating anode X-ray	1249 reflections with *F*^2^ > 2σ(*F*^2^)
Graphite monochromator	*R*_int_ = 0.060
Detector resolution: 10.0 pixels mm^-1^	θ_max_ = 68.1°, θ_min_ = 4.5°
ω–scan	*h* = −7→7
Absorption correction: multi-scan (*ABSCOR*; Higashi, 1995)	*k* = −8→8
*T*_min_ = 0.844, *T*_max_ = 0.947	*l* = −11→11
4941 measured reflections	

### Refinement

**Table:**

Refinement on *F*^2^	Hydrogen site location: inferred from neighbouring sites
Least-squares matrix: full	H atoms treated by a mixture of independent and constrained refinement
*R*[*F*^2^ > 2σ(*F*^2^)] = 0.042	*w* = 1/[σ^2^(*F*_o_^2^) + (0.0317*P*)^2^] where *P* = (*F*_o_^2^ + 2*F*_c_^2^)/3
*wR*(*F*^2^) = 0.088	(Δ/σ)_max_ < 0.001
*S* = 1.04	Δρ_max_ = 0.19 e Å^−^^3^
1533 reflections	Δρ_min_ = −0.19 e Å^−^^3^
121 parameters	Absolute structure: Flack *x* determined using 421 quotients [(*I*^+^)-(*I*^-^)]/[(*I*^+^)+(*I*^-^)] (Parsons *et al.*, 2013)
1 restraint	Absolute structure parameter: 0.08 (19)
Primary atom site location: structure-invariant direct methods	

### Special details

**Table:**

Geometry. All e.s.d.'s (except the e.s.d. in the dihedral angle between two l.s. planes) are estimated using the full covariance matrix. The cell e.s.d.'s are taken into account individually in the estimation of e.s.d.'s in distances, angles and torsion angles; correlations between e.s.d.'s in cell parameters are only used when they are defined by crystal symmetry. An approximate (isotropic) treatment of cell e.s.d.'s is used for estimating e.s.d.'s involving l.s. planes.
Refinement. Refinement of *F*^2^ against ALL reflections. The weighted *R*-factor *wR* and goodness of fit *S* are based on *F*^2^, conventional *R*-factors *R* are based on *F*, with *F* set to zero for negative *F*^2^. The threshold expression of *F*^2^ > σ(*F*^2^) is used only for calculating *R*-factors(gt) *etc*. and is not relevant to the choice of reflections for refinement. *R*-factors based on *F*^2^ are statistically about twice as large as those based on *F*, and *R*- factors based on ALL data will be even larger.

### Fractional atomic coordinates and isotropic or equivalent isotropic displacement parameters (Å^2^)

**Table:**

	*x*	*y*	*z*	*U*_iso_*/*U*_eq_	
O1	−0.5584 (4)	−0.0806 (3)	0.0372 (2)	0.0332 (6)	
O2	0.0502 (3)	0.2156 (3)	−0.0848 (2)	0.0321 (6)	
O3	−0.2569 (3)	0.0470 (3)	−0.05383 (19)	0.0263 (6)	
O4	0.4674 (3)	0.1263 (4)	0.4196 (2)	0.0392 (7)	
O5	0.2469 (3)	0.1713 (4)	0.5922 (2)	0.0364 (6)	
N1	−0.3063 (4)	0.0979 (4)	0.1651 (2)	0.0252 (7)	
H1	−0.379 (5)	0.106 (5)	0.237 (3)	0.038*	
C1	−0.3944 (5)	0.0130 (5)	0.0540 (3)	0.0253 (8)	
C2	−0.0878 (5)	0.1641 (4)	−0.0103 (3)	0.0240 (7)	
C3	−0.1090 (5)	0.2074 (5)	0.1394 (3)	0.0241 (7)	
H3	−0.1422	0.3429	0.1507	0.029*	
C4	0.0989 (5)	0.1562 (5)	0.2255 (3)	0.0266 (7)	
H4A	0.1246	0.0203	0.2191	0.032*	
H4B	0.2290	0.2205	0.1896	0.032*	
C5	0.0798 (5)	0.2093 (6)	0.3733 (3)	0.0338 (9)	
H5A	0.0502	0.3448	0.3790	0.041*	
H5B	−0.0488	0.1430	0.4092	0.041*	
C6	0.2862 (5)	0.1639 (5)	0.4611 (3)	0.0308 (9)	
C7	0.4353 (5)	0.1343 (6)	0.6871 (3)	0.0366 (10)	
H7A	0.3876	0.1435	0.7804	0.044*	
H7B	0.4924	0.0087	0.6714	0.044*	
H7C	0.5531	0.2259	0.6737	0.044*	

### Atomic displacement parameters (Å^2^)

**Table:**

	*U*^11^	*U*^22^	*U*^33^	*U*^12^	*U*^13^	*U*^23^
O1	0.0239 (13)	0.0404 (14)	0.0347 (13)	−0.0065 (12)	−0.0033 (10)	0.0006 (11)
O2	0.0276 (13)	0.0432 (16)	0.0260 (11)	−0.0005 (12)	0.0059 (10)	0.0021 (11)
O3	0.0240 (12)	0.0339 (14)	0.0208 (10)	−0.0010 (11)	−0.0005 (9)	−0.0010 (10)
O4	0.0261 (13)	0.0688 (19)	0.0229 (11)	0.0018 (13)	0.0034 (10)	−0.0013 (12)
O5	0.0275 (13)	0.0629 (17)	0.0185 (10)	0.0037 (13)	−0.0011 (9)	−0.0015 (11)
N1	0.0198 (15)	0.0356 (17)	0.0204 (12)	−0.0033 (12)	0.0017 (11)	−0.0019 (13)
C1	0.0241 (19)	0.0300 (19)	0.0217 (16)	0.0033 (16)	0.0004 (15)	0.0017 (14)
C2	0.0194 (17)	0.0281 (17)	0.0241 (15)	0.0025 (16)	−0.0012 (13)	0.0030 (14)
C3	0.0182 (16)	0.0310 (18)	0.0230 (15)	−0.0013 (15)	0.0012 (13)	0.0005 (15)
C4	0.0191 (16)	0.0379 (19)	0.0225 (15)	−0.0008 (16)	−0.0011 (13)	−0.0011 (14)
C5	0.0232 (19)	0.053 (2)	0.0248 (16)	0.0037 (18)	−0.0017 (14)	−0.0008 (18)
C6	0.0274 (19)	0.042 (2)	0.0234 (16)	−0.0040 (18)	0.0009 (15)	−0.0026 (16)
C7	0.034 (2)	0.054 (3)	0.0203 (16)	0.0033 (19)	−0.0037 (15)	0.0011 (17)

### Geometric parameters (Å, º)

**Table:**

O1—C1	1.196 (3)	C3—C4	1.517 (4)
O2—C2	1.196 (3)	C3—H3	1.0000
O3—C2	1.369 (3)	C4—C5	1.516 (4)
O3—C1	1.403 (3)	C4—H4A	0.9900
O4—C6	1.215 (3)	C4—H4B	0.9900
O5—C6	1.327 (3)	C5—C6	1.508 (4)
O5—C7	1.453 (3)	C5—H5A	0.9900
N1—C1	1.336 (4)	C5—H5B	0.9900
N1—C3	1.457 (4)	C7—H7A	0.9800
N1—H1	0.85 (3)	C7—H7B	0.9800
C2—C3	1.519 (4)	C7—H7C	0.9800
			
C2—O3—C1	109.2 (2)	C3—C4—H4A	109.2
C6—O5—C7	116.4 (2)	C5—C4—H4B	109.2
C1—N1—C3	113.1 (2)	C3—C4—H4B	109.2
C1—N1—H1	121 (2)	H4A—C4—H4B	107.9
C3—N1—H1	124 (2)	C6—C5—C4	113.1 (3)
O1—C1—N1	130.9 (3)	C6—C5—H5A	109.0
O1—C1—O3	120.6 (3)	C4—C5—H5A	109.0
N1—C1—O3	108.5 (3)	C6—C5—H5B	109.0
O2—C2—O3	121.7 (3)	C4—C5—H5B	109.0
O2—C2—C3	129.1 (3)	H5A—C5—H5B	107.8
O3—C2—C3	109.2 (2)	O4—C6—O5	123.2 (3)
N1—C3—C4	115.2 (3)	O4—C6—C5	125.4 (3)
N1—C3—C2	99.9 (2)	O5—C6—C5	111.4 (3)
C4—C3—C2	112.5 (2)	O5—C7—H7A	109.5
N1—C3—H3	109.6	O5—C7—H7B	109.5
C4—C3—H3	109.6	H7A—C7—H7B	109.5
C2—C3—H3	109.6	O5—C7—H7C	109.5
C5—C4—C3	111.9 (2)	H7A—C7—H7C	109.5
C5—C4—H4A	109.2	H7B—C7—H7C	109.5
			
C3—N1—C1—O1	−176.9 (3)	O2—C2—C3—C4	−56.0 (5)
C3—N1—C1—O3	4.0 (4)	O3—C2—C3—C4	123.0 (3)
C2—O3—C1—O1	177.2 (3)	N1—C3—C4—C5	−69.4 (4)
C2—O3—C1—N1	−3.6 (3)	C2—C3—C4—C5	177.1 (3)
C1—O3—C2—O2	−179.0 (3)	C3—C4—C5—C6	−178.8 (3)
C1—O3—C2—C3	1.9 (3)	C7—O5—C6—O4	0.9 (5)
C1—N1—C3—C4	−123.4 (3)	C7—O5—C6—C5	−178.6 (3)
C1—N1—C3—C2	−2.7 (3)	C4—C5—C6—O4	15.1 (5)
O2—C2—C3—N1	−178.6 (3)	C4—C5—C6—O5	−165.5 (3)
O3—C2—C3—N1	0.4 (3)		

### Hydrogen-bond geometry (Å, º)

**Table:**

*D*—H···*A*	*D*—H	H···*A*	*D*···*A*	*D*—H···*A*
N1—H1···O4^i^	0.86 (3)	2.07 (3)	2.926 (3)	176 (3)
C7—H7*A*···O2^ii^	0.98	2.54	3.366 (4)	142

Symmetry codes: (i) *x*−1, *y*, *z*; (ii) *x*, *y*, *z*+1.
